# Relationship Between T-Cell Exosomes and Cellular Subsets in SLE According to Type I IFN-Signaling

**DOI:** 10.3389/fmed.2020.604098

**Published:** 2020-11-30

**Authors:** Patricia López, Javier Rodríguez-Carrio, Luis Caminal-Montero, Ana Suárez

**Affiliations:** ^1^Department of Functional Biology, Immunology Area, Faculty of Medicine, University of Oviedo, Oviedo, Spain; ^2^Group of Basic and Translational Research in Inflammatory Diseases, Instituto de Investigación Sanitaria del Principado de Asturias (ISPA), Oviedo, Spain; ^3^Department of Internal Medicine, Hospital Universitario Central de Asturias, Oviedo, Spain

**Keywords:** exosomes, senescent CD4^+^CD28^null^ T cells, systemic lupus erythematosus, type I interferon (IFN), cytokines

## Abstract

**Objective:** To quantify the levels of circulating exosomes derived from T-cells and monocytes and their possible associations with leukocyte subpopulations and cytokine milieu in Systemic Lupus Erythematosus (SLE).

**Methods:** Total circulating exosomes (CD9^+^-Ex) and those derived from T-cells (CD3^+^-Ex) and monocytes (CD14^+^-Ex) were quantified by flow cytometry in 82 SLE patients and 32 controls. Leukocyte subsets and serum cytokines were analyzed by flow cytometry or by immunoassays. IFN-score was evaluated by real time RT-PCR in whole blood samples from a subgroup of 73 patients and 24 controls.

**Results:** Activation markers (IFNR1 and BLyS) on monocytes, neutrophils and B-cells correlated inversely with circulating exosomes (CD9^+^-Ex, CD3^+^-Ex, and CD14^+^-Ex) in controls but directly with CD3^+^-Ex in patients (all *p* < 0.05). Although CD9^+^-Ex were increased in SLE, no differences were found in CD3^+^-Ex, supporting that exosome content accounts for this opposite role. Interestingly, CD4^+^CD28^null^ cells correlated with CD3^+^-Ex in patients and controls, and displayed similar associations with leukocyte subsets in both groups. Additionally, CD3^+^-Ex correlated in patients with the expression of CD25 in CD4^+^CD28^null^ cells. Furthermore, the activated status of this senescent subset was related to IFNα serum levels in controls and to IFN-score in SLE patients. Finally, patients presenting high IFN-score, in addition to elevated CD25^+^CD28^null^ cells associated with the activation of myeloid cells, displayed higher levels of inflammatory cytokines and chemokines.

**Conclusion:** Our results support a relationship between T-cell exosomes and cellular subsets in SLE according to type I IFN-signaling, which could amplify chronic immune activation and excessive cytokine/chemokine response.

## Introduction

Systemic lupus erythematosus (SLE) is a chronic autoimmune disease with multiple clinical manifestations and numerous cellular and molecular abnormalities in the immune system, including autoantibody production and leukocyte activation (1). Actually, SLE patients display a generalized immune stimulation characterized by the presence of over-activated and senescent lymphocytes, as well as by altered myeloid subpopulations, such as monocytes, dendritic cells (DC), and neutrophils (2, 3). Despite the unknown etiology of SLE, several lines of evidence emphasize the role of the crosstalk among different immune cells as a key mediator of the systemic inflammation and organ damage characteristic of lupus pathogenesis. Altered production of cytokines has largely been considered as the main responsible of cellular communication. However, cytokine blockade in lupus has yielded controversy results, hence suggesting the involvement of other mediators. Therefore, the role of extracellular vesicles has recently emerged as effective vehicles, along with cytokines, of intercellular signaling in physiological and pathological situations (4).

Exosomes are endosomal multivesicular bodies (30–150 nm) formed after fusion with the plasma membrane in many cell types. They can be found in several corporal fluids and carry cell-specific content of bioactive molecules, such as of proteins, lipids, and nucleic acids (5), thus acting as a “fingerprint” of the phenotypic and physiological state of the cell of origin upon exosome shedding. Therefore, cell specific exosomes could serve as a potential signature in pathological conditions. Accordingly, it has been observed that levels of circulating exosomes correlate with disease activity in lupus patients, thus representing potential biomarkers in SLE (6).

Although the biological functions of exosomes are not fully understood, it is known that exosomes can be selectively transferred to target cells to modulate many cellular processes, including immune response. Thus, after release of the exosome cargo in immune cells, both immunostimulatory and regulatory roles have been reported (7, 8). In the context of autoimmunity, and SLE in particular, extracellular vesicles could also represent a source of self-antigens (9) able to participate in the formation of immune-complexes, amplification of autoreactive T-cells activation (10, 11) and secretion of pro-inflammatory cytokines (12).

Among cytokines involved in SLE, type I IFNs plays a central role (13), since IFNα serum levels or expression of IFN-inducible genes are increased in most patients and usually correlated with disease activity and clinical manifestations (14). Binding of IFNα to the type I IFN receptor (IFNAR) on B and T cells, monocytes, macrophages, DCs, and neutrophils stimulates immunoregulatory effects in these cells (15). Thus, IFNα is able to induce the expression of other pathogenic cytokines in SLE, such as BLyS and IL-17, acting in a pathological axis that perpetuates the inflammation and disease progression (2, 16, 17).

In this scenario, the presence of “SLE-specific” exosomes derived from over-activated leukocytes may promote the vicious circle of immune dysregulation. However, the cellular source of exosomes and their immunomodulatory role in SLE are not well-understood. In the current study we quantified the circulating levels of total, T-cell, and monocyte derived exosomes and their possible associations with several leukocyte subsets usually altered in SLE. In addition, the role of IFNα and other relevant cytokines was analyzed.

## Patients and Methods

### Patients and Controls

Eighty-two SLE patients fulfilling the American College of Rheumatology (ACR) revised criteria for the SLE classification were sequentially recruited from the outpatient clinic of the Autoimmune Disease Unit (Hospital Universitario Central de Asturias, HUCA). Information on clinical manifestations along the disease course was obtained after a retrospective review of their clinical records, whereas parameters of disease activity (anti-dsDNA titer and SLE disease activity index, SLEDAI) and treatments received over the previous 3 months were recorded at the sampling time (Table 1). Thirty-two volunteer without any pathology or treatment were recruited as healthy controls (C). Ethical approval for this study was obtained from the Regional Ethics Committee for Clinical Research (Servicio de Salud del Principado de Asturias), according to the Declaration of Helsinki. Written informed consent was signed from all individuals prior to participation in the study.

**Table 1:** Demographic and clinical features of SLE patients.

	**SLE patients (*n =* 82)**	**Controls (*n =* 32)**
**Demographic features**		
Sex, *n* (female/male)	75/6	24/8
Age, years (mean ± SD)	49.17 ± 12.08	48.49 ± 6.72
**Leukocyte counts**, **× 10**^**3**^ **cells/ul (mean ± SD)**		
Total leukocytes	4.91 (2.51)^a^***	6.13 (2.19)
Lymphocytes	1.45 ± 0.63***	2.26 ± 0.49
Monocytes	0.41 ± 0.16	0.48 ± 0.16
Neutrophils	3.20 ± 1.70*	3.66 ± 1.61
**Clinical manifestations**, ***n*** **(%)**		
Age at diagnosis, years (mean ± SD)	34.89 ± 13.66	
Disease duration, years (mean ± SD)	13.96 ± 10.25	
SLEDAI score (mean ± SD)	3.76 ± 4.10	
C3, g/l (mean ± SD)	0.91 ± 0.30	
C4, g/l (mean ± SD)	0.18 ± 0.09	
ACR criteria		
Malar rash	43 (52.44)	
Discoid lesions	20 (24.39)	
Photosensitivity	45 (54.88)	
Oral ulcers	42 (51.22)	
Arthritis	57 (69.51)	
Serositis	17 (20.63)	
Cytopenia	56 (68.29)	
Renal disorder	23 (28.05)	
Neurological disorder	8 (9.76)	
**Autoantibodies**, ***n*** **(%)**		
ANAs	82 (100.00)	
Anti-dsDNA/titer,U/ml (mean ± SD)	66 (80.49)/37.76 ± 63.29	
Anti-SSA	42 (51.22)	
Anti-SSB	14 (17.07)	
Anti-Sm	6 (7.32)	
Anti-RNP	11 (13.41)	
Rheumatoid factor	12 (14.63)	
Anti-cardiolipin IgG/IgM	14 (17.28)	
**Treatment**, ***n*** **(%)**		
None or NSAIDs	3 (3.66)	
Antimalarial drugs	72 (87.80)	
Glucocorticoids	33 (40.24)	
Immunosuppressive drugs^b^	2 (2.44)	

*dsDNA, double stranded DNA; RF, rheumatoid factor; NSAID, non-steroidal anti-inflammatory drug*.

a Differences between patients and controls were evaluated by Test U-Mann-Whitney (*p < 0.05; ****p < 0.001).

b *Mycophenolate mophetil, azathioprine*.

### Exosome Isolation and Quantification by Flow Cytometry

Total and subset-specific circulating exosomes were detected by flow cytometry in serum samples from SLE patients and C by using the *ExoStep*™ kit (Immunostep) following the manufacturer's recommendations (18). Briefly, serum samples from patients and controls were centrifuged at 3,000 g for 15 min at room temperature (RT). Then, supernatants were incubated overnight at RT with capture beads (polystyrene micro-particles coated with antibodies against CD63 and with discrete fluorescence intensity). Later, bead-bound exosomes were washed with PBS 1% bovine serum albumin (BSA) and collected after centrifugation at 2,500 g-5 min-RT. Bead-bound exosomes were labeled by incubation (60 min, at 4°C in darkness) with primary antibodies against tetraspanins or specific lineage markers conjugated with biotin. Thus, total exosomes were identified by using anti-CD9, exosomes from T-cells by anti-CD3, and exosomes from monocytes by anti-CD14 antibodies (all from Immunostep). Also, additional tubes without exosomes were prepared for background determination. Samples were washed with PBS 1% BSA and centrifuged at 2,500 g-5 min-RT. Finally, exosomes were incubated (30 min, at 4°C in darkness) with streptavidin-PE (Immunostep), washed (2,500 g-5 min-RT) and resuspended in 350 μL of PBS. Then, 2,000 microbeads were immediately acquired using a FACS Canto II flow cytometer (BD). Bead-exosomes population was gated according to their own fluorescence on FL3 vs. FL4 channels and the quantity of exosomes was determined by the MFI (mean fluorescence intensity) in the FL2 channel. Relative Fluorescence Intensity (RFI) was calculated as MFI positive/MFI background.

### Flow Cytometry Analysis of Fresh Blood Cells

Peripheral blood samples from SLE patients and controls were collected in ethylene diamine tetra-acetic acid (EDTA)-containing tubes as anticoagulant. BLyS-FITC (eBiosciences) and IFNRA1-PE (R&D Systems) expression was simultaneously quantified on monocytes, B cells, and neutrophils. Monocytes and B cells were identified by expression of CD14-APC-Cy7 (BioLegend), or CD19-CF-Blue (Immunostep), respectively, while neutrophils were identified according to their distinctive forward and side-scatter signals, as previously described (2). To quantify activated CD4^+^ T-cell subsets (no-Treg CD25^+^), blood samples were stained with anti-CD4-APC-Cy7, anti-CD25-FITC, and anti-CD127-PE-Cy7 antibodies (all from eBiosciences). Afterward, blood samples were lysed with 2 ml of BD Lysing Solution (BD Biosciences) for 5 min and washed twice with PBS. Then, cells were fixed, permeabilized and intracellularly stained with anti-FOXP3-PE (*Foxp3/transcription factor staining buffer set*; eBiosciences) to determine the Treg population. The amount of Treg cells was subtracted from the total CD25^+^CD4^+^ to obtain activated CD4^+^ T-lymphocytes. Circulating senescent T-cells (CD4^+^CD28^null^) were identified by staining with anti-CD3-PerCP-Cy5.5 (Tonbo Biosciences), anti-CD4-Pacific-Blue (Immunostep), anti-CD25-PE (Immunostep), and anti-CD28-APC-Cy7 (BD Biosciences) antibodies. Isotype fluorochrome-matched control antibodies (eBioscence, USA) were used as negative controls for the cytometry analysis. Cells were stained for 30 min at 4°C with the appropriate monoclonal antibody, washed twice and re-suspended in PBS. Acquisition of 30,000–200,000 events/tube was performed using a FACS Canto II flow cytometer (BD). The analysis was based on cells located the area of dots termed “the living region” which was defined using forward and side scatter. Samples were subsequently analyzed using FlowJo software (Scripps Research Institute). Results were expressed as the percentage of positive cells or MFI.

### Cytokine Quantification

Serum samples from patients and controls were maintained at −80°C until cytokine determinations. IFNα, IL-17A, IL-10, IL-6, CCL3 (MIP-1α), and CCL5 (RANTES) concentrations were quantified by Cytometric Bead Arrays Flex Set by using a FACS Canto II flow cytometer (BD) and following the manufacturer's instructions. For IL-10 and IL-6, an Enhanced Sensitivity Flex Set was used. Finally, ELISA kits were used for the quantification of TNFα, CCL2 (MCP-1), CXCL10 (IP-10) (Mini kit, PeproTech), IFNγ (OptEIA kit, BD) and BLyS (Human BAFF Instant ELISA, eBioscience) following the manufacturer's instructions. The lower limits of detection were 1.25 pg/ml for IFNα, 0.30 pg/ml for IL-17A, 0.014 pg/ml for IL-10 and IL-6, 0.20 pg/ml for CCL3, 0.002 pg/ml for CCL5, 3.90 pg/ml for TNFα, 8.00 pg/ml for CCL2, 16.00 pg/ml for CXCL10, 0.58 pg/ml for IFNγ, and 0.13 ng/ml for BLyS.

### Gene Expression Assays

Blood samples were immediately mixed with *RNA Stabilization Reagent for Blood/Bone Marrow* (Roche) and stored at −20°C. Next, samples were thawed at RT and mRNA was isolated using the *mRNA Isolation Kit for Blood/Bone Marrow* (Roche) and following the manufacturer's instructions. Reverse transcription was performed using a *High-Capacity cDNA Reverse Transcription Kit* (Applied Biosystems). Gene expression was evaluated in 73 SLE patients and 24 C by Real-Time PCR with pre-designed *TaqMan Gene Expression Assays* (Applied Biosystems, Germany) for the following interferon regulated genes (IRGs): IFI44 (interferon-induced protein 44, reference Hs00197427_m1), IFI44L (interferon induced protein 44 like, reference Hs00915292_m1), MX1 (MX dynamin like GTPase 1, reference Hs00895608_m1), and IFI6 (interferon-alpha inducible protein 6, reference Hs00242571_m1). Reactions were performed in *TaqMan Gene Expression Master Mix* (Applied Biosystems). Real-Time quantitative PCR was performed in an ABI Prism HT7900 instrument (Applied Biosystems) and Ct values were analyzed with the software SDS 2.3. All samples were assayed by triplicate and the average was used. Expression level was evaluated by the 2^−ΔCt^ method, using the GAPDH gene expression (glyceraldehyde-3-phosphate dehydrogenase, reference Hs99999905_m1) as housekeeping to normalize Ct values. The expression levels were log-transformed and individual Z-scores were calculated for each gene from the distribution observed in the whole population. All Z-scores were used to determine the composite IFN-score.

### Statistical Analysis

Variables were summarized as median (interquartile range, IQR) unless otherwise stated. The Kolmogorov-Smirnov test was used to assess the normal distribution of the data. Differences were assessed by Mann–Whitney *U* or *T*-tests as appropriated. Correlations were analyzed by Spearman's rank test and confirmed by multivariate regression analysis. A *p* < 0.05 was considered statistically significant. Data were analyzed using GraphPad Prism 8 software (GraphPad Software) and SPSS 24 statistical software package (SPSS Inc.).

## Results

### Exploring the Interactions Between T-Cell and Monocyte-Derived Exosomes and Leukocyte Subsets in SLE

**Figure 1:**
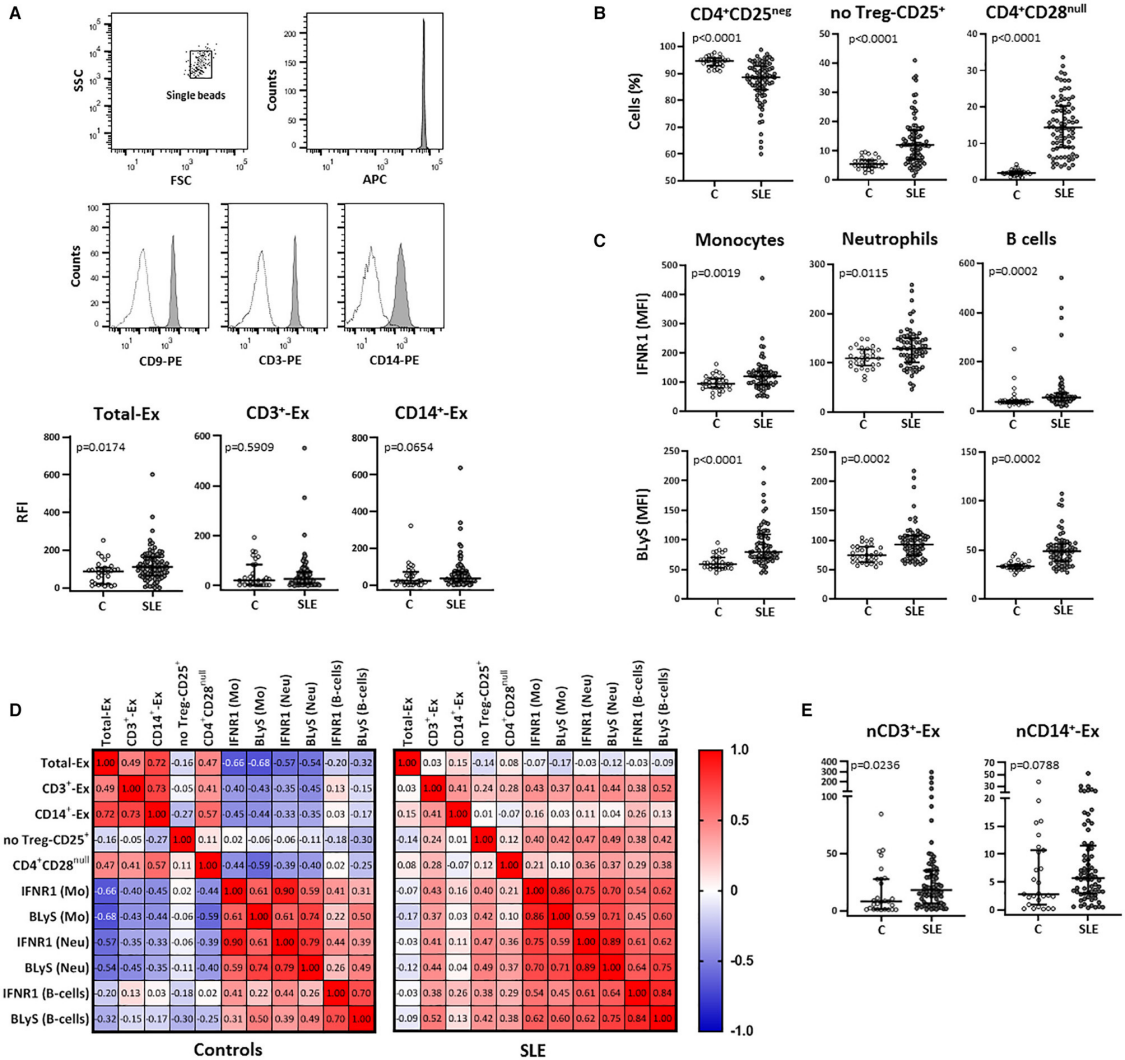
Total and T-cell and monocyte derived circulating exosomes and their relationship with cellular subsets in SLE patients and controls. Total exosomes (CD9^+^- Ex) and those derived from T-cells (CD3^+^-Ex) and monocytes (CD14^+^-Ex), along with several blood cellular subsets, were quantified by flow cytometry in serum samples from SLE patients (SLE) and healthy controls **(C)**. **(A)** Exosomes immobilized on APC-beads were stained using biotinylated antibody followed by PE-conjugated streptavidin and quantified by flow cytometry. A gate containing single beads was created according to the forward and side scatter (FSC and SSC, respectively) plot, and another gate containing single beads confirmed the APC fluorescence of microbeads (representative dot plots of a SLE patient are shown). Histograms represent CD9–, CD3–, and CD14-PE expression in a SLE patient as an example (shaded) with the respective negative control (dotted line). Scatter plots display RFI values of CD9^+^-, CD3^+^-, and CD14^+^-exosomes from SLE and C. **(B)** Graphs represent percentage of CD4^+^CD25^neg^, no Treg-CD25^+^ and CD4^+^CD28^null^ cells in SLE and C. **(C)** IFNR1 and BLyS levels on surface of monocytes, neutrophils and B-cells from patients and controls. **(D)** Correlation matrices among different types of circulating exosomes and cellular subsets in SLE and controls, where the color of the tiles is proportional to the strength of the correlation between each pair of variables and the numbers represented in the correlograms are the ρ-coefficients (Spearman tests). **(E)** Amounts of CD3^+^- and CD14^+^-exosomes normalized respect to the absolute T-cell (nCD3^+^-Ex) and monocyte (nCD14^+^-Ex) counts in patients and controls. Horizontal lines represent median and interquartile range of RFI values of exosomes in **(A)**, percentage of cells in **(B)**, MFI levels of surface markers in **(C)**, and normalized amounts of exosomes subsets (RFI values of CD3^+^ or CD14^+^-exosomes/absolute T-cells or monocytes, respectively) in **(E)**; statistical differences among groups were evaluated by Mann-Whitney *U*-test.

Total exosomes (CD9^+^-Ex) were elevated in serum from SLE patients compared to controls and a similar trend was observed in monocyte-derived exosomes (CD14^+^-Ex), whereas no differences were found for those T-cell-derived (CD3^+^-Ex) (Figure 1A). Exosome levels were not associated with demographic or clinical parameters, and no differences were detected between active (SLEDAI≥7) and non-active patients in any subset (all *p* > 0.05).

The analysis of blood cellular subsets revealed higher levels of activated and senescent CD4^+^ lymphocytes (no-Treg CD25^+^ and CD28^null^, respectively) in SLE patients compared to controls, whereas resting T-cells were decreased (Figure 1B) and an increase was observed for FOXP3^+^ Treg cells [1.34 (0.80) vs. 0.91 (1.27); *p* = 0.010]. Also, the increased expression of IFNR1 and BLyS observed in monocytes, neutrophils, and B-cells from SLE, supports a higher activation status of these leukocyte subsets (Figure 1C).

Correlation analyses between exosomes and cellular subsets revealed different, and even opposite, findings in controls and patients (Figure 1D). In healthy controls, exosome levels (CD9^+^-Ex, CD3^+^-Ex, and CD14^+^-Ex) were negatively associated with the activation of monocytes and neutrophils, whereas a positive correlation of CD3^+^-Ex with the activation markers of myeloid cells and lymphocytes (all *p* < 0.05) was observed in SLE. These associations were maintained after adjusting for sex, age, disease activity, and duration in a linear regression analyses model (Table 2).

**Table 2:** Relationship between CD3^+^-Ex and cellular subsets in SLE patients.

	**B (95% CI)**	***P*-value**	***R***
No Treg-CD25^+^	0.714 (0.268, 1.160)	**0.002**	0.402
CD4^+^CD28^null^	0.716 (0.182, 1.250)	**0.009**	0.369
IFNR1 (monocytes)	0.819 (0.084, 1.155)	**0.030**	0.363
BlyS (monocytes)	0.694 (0.010, 1.377)	**0.047**	0.346
IFNR1 (neutrophils)	1.311 (0.230, 2.392)	**0.018**	0.380
BlyS (neutrophils)	2.073 (0.835, 3.310)	**0.001**	0.460
IFNR1 (B-cells)	0.687 (0.159, 1.215)	**0.012**	0.396
BlyS (B-cells)	1.363 (0.637, 2.088)	** <0.001**	0.494

*Multivariate lineal regression adjusted for sex, age, SLEDAI, and disease duration. Bold values represent statistically significant ones (p < 0.05)*.

Therefore, the activated and senescent status of T-cells present in SLE patients could generate CD3^+^-Ex with different effects than those derived from healthy T-cells. However, whereas circulating levels of CD3^+^-Ex levels were unchanged, T-cell number was significantly reduced in patients [2.22 (0.75) vs. 1.32 (0.77) × 10^3^ cells/μl, *p* < 0.001]. Actually, after normalizing exosome levels with absolute T-cell and monocyte counts, a significant increase of CD3^+^-Ex was observed in SLE patients compared with controls (Figure 1E).

Curiously, in spite of the lack of differences between patients and controls, CD3^+^-Ex levels were directly correlated in both groups with CD4^+^CD28^null^ cells (*p* < 0.05), a population strikingly increased in SLE. Moreover, CD4^+^CD28^null^ cells showed a similar behavior than CD3^+^-Ex, displaying negative correlations with most activated cell populations in controls, but being positive in SLE patients. Furthermore, CD3^+^-Ex were correlated in patients with the CD25 expression in CD4^+^CD28^null^ cells (ρ = 0.261, *p* = 0.031), independently of disease activity and duration, sex and age [B (95% CI): 1.516 (0.150, 2.882), *p* = 0.030]. In fact, CD25 expression was significantly increased in CD4^+^CD28^null^ cells from SLE patients compared to controls (*p* = 0.008). Accordingly, the SLE group displayed an augmented proportion of CD4^+^CD28^null^ cells expressing CD25 (CD25^+^CD28^null^) compared with controls (9.77 vs. 5.95%, *p* = 0.002). Interestingly, the percentage of CD25^+^CD28^null^ cells in controls was related to IFNα serum levels (ρ = 0.369, *p* = 0.038), thus suggesting that a continuous IFN-signaling could be involved in the activation of senescent-T cells in SLE.

All these results support that qualitative rather than quantitative differences characterize SLE exosomes, especially those derived from T-cells. The activation of senescent CD4^+^CD28^null^ lymphocytes and the IFNα pathway could play a role in this scenario.

### Influence of IFN-Signaling on Exosomes Derived From T Cells

**Figure 2:**
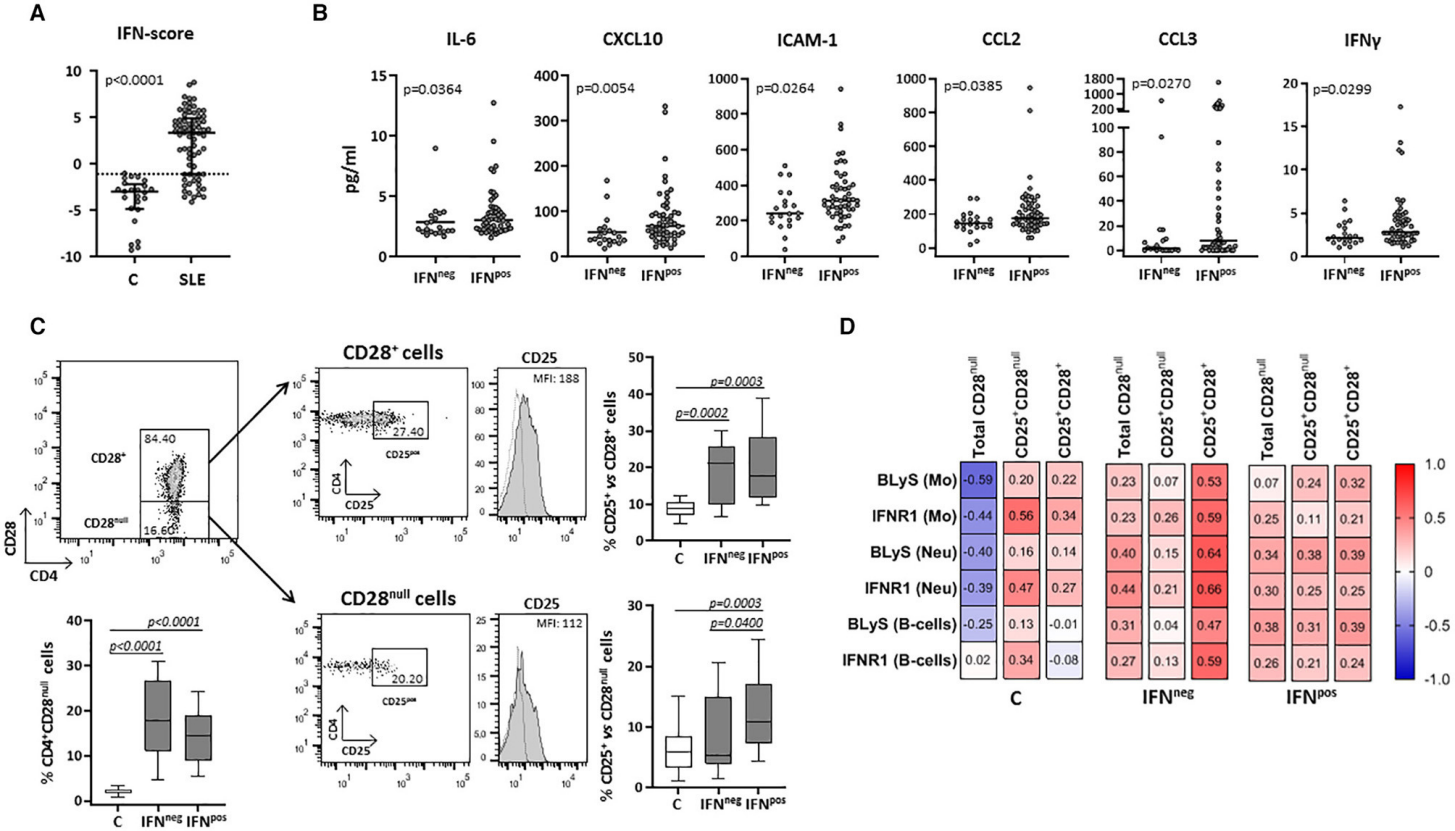
Circulating levels of cytokines and cellular subsets in controls and SLE patients according to their IFN-score. The expression of four IRGs was quantified in blood samples from SLE patients (SLE) and healthy controls (C). **(A)** Scatter plots display IFN-score in SLE and C. Dotted line represents the P^90^ of IFN-score in controls, employed to classify SLE patients in IFN^neg^ or IFN^pos^ individuals. **(B)** Serum levels of IL-6, CXCL10, ICAM-1, CCL2, CCL3, and IFNγ in IFN^neg^ vs. IFN^pos^ patients. **(C)** Total CD4^+^CD28^null^ and activated conventional or senescent CD4^+^ T-cells (CD25^+^CD28^+^ and CD25^+^CD28^null^, respectively) in IFN^neg^ and IFN^pos^ SLE groups compared with controls. Representative dot plots of CD4 vs. CD28 expression in gated T-cells from a SLE patient are shown. Histograms represent CD25 expression in a SLE patient as an example (shaded) with the respective isotype matched control antibody (dotted line). Numbers in plots indicate the median fluorescence intensity (MFI) with the matched irrelevant control value subtracted (histograms) or percentage of gated cells (dot-plots). Horizontal lines represent median and interquartile range; statistical differences among groups were evaluated by Mann-Whitney *U*-test. **(D)** Correlations among activated subsets of CD4^+^ T-cells and myeloid populations in IFN^neg^/IFN^pos^ patients and controls, where the color of the tiles is proportional to the strength of the correlation between each pair of variables and the numbers represented in the correlograms are the ρ-coefficients (Spearman tests).

The expression of four IRGs was quantified in patients and controls and the composite IFN-score was calculated (Figure 2A). As expected, the IFN-score was strikingly increased in most of patients, and allowed their classification as IFN^neg^ or IFN^pos^ individuals (P^90^ in controls). The IFN^pos^ group (72.60% of patients) was more likely to exhibit ENAs (extractable nuclear antigens) positivity, particularly SSA and RNP, lower complement levels and earlier diagnosis, whereas no differences were observed for the rest of clinical features (Table 3). Also, the quantification of circulating cytokines (Table 4) revealed increased levels of IL-6, IFNγ, ICAM-1, CXCL10, CCL2, and CCL3 in IFN^pos^ patients compared to their IFN^neg^ counterparts (Figure 2B), whereas IL-10, BLyS, TNFα, and IFNα amounts were elevated in patients irrespective to IFN status.

**Table 3:** Demographic and clinical features of IFN^pos^ *vs* IFN^neg^ SLE patients.

	**IFN^**pos**^ (*n =* 53)**	**IFN^**neg**^ (*n =* 20)**	***P*-value^a^**
**Demographic and clinical features**			
Sex, *n* (female/male)	50/3	18/2	0.709
Age, years (mean ± SD)	46.33 ± 10.95	54.36 ± 12.45	**0.023**
Age at diagnosis, years (mean ± SD)	31.91 ± 12.26	41.00 ± 13.03	**0.010**
Disease duration, years (mean ± SD)	14.43 ± 9.54	12.70 ± 12.42	0.284
SLEDAI score (mean ± SD)	3.91 ± 3.93	2.65 ± 3.28	0.132
C3, g/l (mean ± SD)	0.88 ± 0.23	1.07 ± 0.40	0.084
C4, g/l (mean ± SD)	0.16 ± 0.05	0.23 ± 0.14	**0.036**
ACR criteria			
Malar rash	29 (54.72)	10 (50.00)	0.672
Discoid lesions	12 (22.64)	4 (20.00)	0.680
Photosensitivity	29 (54.72)	11 (55.00)	0.505
Oral ulcers	31 (58.49)	8 (40.00)	0.437
Arthritis	35 (66.04)	14 (70.00)	0.874
Serositis	10 (18.87)	4 (20.00)	0.862
Cytopenia	37 (69.81)	13 (65.00)	0.931
Renal disorder	17 (32.07)	3 (15.00)	0.270
Neurological disorder	5 (9.43)	3 (15.00)	0.696
**Autoantibodies**, ***n*** **(%)**			
ANAs	53 (100.00)	20 (100.00)	-
Anti-dsDNA/titer, U/ml (mean ± SD)	43 (81.13)/33.26 ± 41.94)	16 (80.00)/30.40± 64.41	0.352/0.338
ENAs	39 (73.58)	8 (40.00)	**0.018**
Anti-SSA	34 (64.15)	6 (30.00)	**0.005**
Anti-SSB	11 (20.75)	2 (10.00)	0.152
Anti-Sm	5 (9.43)	0 (0.00)	0.238
Anti-RNP	10 (18.87)	0 (0.00)	**0.005**
Rheumatoid factor	9 (16.98)	3 (15.00)	0.876
Anti-cardiolipin IgG/IgM	8 (15.09)	4 (20.00)	0.665
**Treatment**, ***n*** **(%)**			
None or NSAIDs	1 (1.89)	1 (5.00)	0.922
Antimalarial drugs	47 (88.68)	17 (85.00)	0.976
Glucocorticoids	20 (37.74)	8 (40.00)	0.606
Immunosuppressive drugs^b^	1 (1.89)	2 (10.00)	0.688

*dsDNA, double stranded DNA; RF, rheumatoid factor; NSAID, non-steroidal anti-inflammatory drug*.

a *Differences were analyzed by χ2 or Mann–Whitney U tests for categorical or continuous variables, respectively*.

b *Mycophenolate mophetil, azathioprine. Bold values represent statistically signifcant ones (p < 0.05)*.

**Table 4:** Serum levels of inflammatory cytokines on SLE patients and healthy controls.

**Molecules**	**Controls (*N =* 32)**	**SLE patients (*N =* 82)**	***P*-value**
IL-6	1.77 (3.24)	2.77 (1.44)	** <0.001**
IL-10	0.22 (1.12)	1.41 (0.47)	** <0.001**
IFNα	2.86 (1.30)	8.53 (8.72)	** <0.001**
IL-17A	3.49 (10.11)	4.50 (12.59)	0.822
TNFα	42.64 (116.17)	164.98 (125.50)	** <0.001**
IFNγ	3.14 (3.10)	2.72 (2.51)	0.149
BLyS	490.11 (154.40)	1887.56 (782.77)	** <0.001**
ICAM-1	255.56 (215.43)	286.03 (160.99)	0.284
CXCL10 (IP-10)	48.30 (36.24)	58.33 (57.33)	** <0.001**
CCL2 (MCP-1)	204.97 (164.63)	167.48 (109.15)	0.388
CCL3 (MIP1α)	0.20 (0.00)	6.44 (51.12)	** <0.001**
CCL5 (RANTES)	20.78 (12.66)	16.01 (12.21)	0.083

*Values represent median (interquartile range) (pg/ml)*.

*Differences analyzed by U-Mann-Whitney test. Bold values represent statistically signifcant ones (p < 0.05)*.

Total CD4^+^CD28^null^ and activated conventional CD4^+^ T-cells (CD25^+^CD28^+^) were increased in both IFN^neg^ and IFN^pos^ SLE groups compared with controls (Figure 2C). However, IFN^pos^ patients exhibited an increased proportion of CD25^+^CD28^null^ cells compared with controls and the IFN^neg^ group, thus supporting a role of IFN-signaling in the senescent-T cells activation.

Interestingly, CD25^+^CD28^null^ cells were positively associated with the expression of activation markers (especially IFNR1) on monocytes, neutrophils and B-cells from controls, in spite of the opposite effect exerted by the total CD4^+^CD28^null^ subset (Figure 2D). In SLE, total CD4^+^CD28^null^ cells were associated with the activation of myeloid cells and lymphocytes in both IFN^neg^ and IFN^pos^ groups, but only in the latter the CD25^+^CD28^null^ subset accounted for this phenomenon (Figure 2D). Moreover, this activated senescent subset was strongly associated with CCL3 (ρ = 0.522, *p* < 0.001) and IL-17 (ρ = 0.455, *p* = 0.001) serum levels in the IFN^pos^ patient group.

On the other hand, in IFN^pos^ patients, but not in those IFN^neg^, CD3^+^-Ex were positively correlated with the size of CD4^+^CD28^null^ and no-Treg CD25^+^ subsets as well as with the CD25 expression in CD4^+^CD28^null^ cells (Table 5). However, CD3^+^-Ex levels showed a no significant reduction in IFN^pos^ compared with IFN^neg^ patients [22.77 (47.03) vs. 41.73 (51.35)]. Actually, multivariate linear regression model including sex, age, disease duration, and activity showed a negative association between IFN-score and CD3^+^-Ex, disease duration presenting a significant positive effect (Table 6).

**Table 5:** Correlation between CD3^+^-Ex levels and T-cell subsets in healthy controls and SLE patients depending on type I IFN status.

	**No-Treg CD25^**+**^ (%)**	**CD4^**+**^CD28^**null**^ (%)**	**CD25 in CD4^**+**^CD28^**null**^ (MFI)**
Controls	ρ = −0.046 *p* = 0.818	***ρ*** **= 0.411** ***p*** **= 0.033**	ρ = −0.118 *p* = 0.536
SLE- IFN^neg^	ρ = 0.211 *p* = 0.387	ρ = 0.193 *p* = 0.429	ρ = 0.291 *p* = 0.274
SLE- IFN^pos^	***ρ*** **= 0.324** ***p*** **= 0.019**	***ρ*** **= 0.399** ***p*** **= 0.004**	***ρ*** **= 0.321** ***p*** **= 0.028**

*Spearman rank correlation test. Bold values represent statistically signifcant ones (p < 0.05)*.

**Table 6:** IFN score association with CD3^+^-Ex levels in SLE patients.

	**B (95% CI)**	***p*-value**
Sex	−1.358 (−4.969, 2.252)	0.455
Age	−4.157 (−12.081, 3.768)	0.298
SLEDAI	1.086 (−1.488, 3.659)	0.402
Disease duration	**1.828 (0.099, 3.558)**	**0.039**
Total-Ex	−0.714 (−3.016, 1.589)	0.538
CD3^+^-Ex	–**1.671 (**–**3.185**, –**0.157)**	**0.031**
CD14^+^-Ex	1.182 (−0.463, 3.422)	0.133

*Multivariate lineal regression. Dependent variable: IFN score (R = 0.380). Bold values represent statistically signifcant ones (p < 0.05)*.

Taken together, these results suggest again the existence of qualitative differences among circulating CD3^+^-Ex depending on the status of parental T-cells, and point out IFN-signaling and activation of senescent CD4^+^ T-cells as underlying factors of the chronic immune activation and excessive cytokine/chemokine response occurring in SLE, aberrant CD3^+^-Ex being significant mediators.

## Discussion

A compelling body of evidence highlights exosomes as mediators of intercellular communication that could be effectively involved in the establishment, perpetuation and modulation of autoimmune diseases (19). However, almost nothing is known about the presence and possible role of exosomes derived from immune cells in these patients. To the best of our knowledge, the present work quantifies for the first time the circulating exosomes derived from T-cells and monocytes in SLE patients and evaluates their clinical relevance. The results herein reported revealed new findings that suggest the participation of exosomes, especially those derived from T-cells, in the activated status of several blood cells, with a pivotal role of senescent CD4^+^CD28^null^ lymphocytes. Furthermore, present findings allow us to propose exosomes as relevant players for the effects of type-I IFN pathway in this scenario.

Our results are in line with the idea that circulating exosomes may exert different effects on the immune system depending on physiopathological conditions, ranging from suppressors in tumors to immunostimulators in chronic inflammatory disorders, as a mirror of the altered systemic immune response (6, 20–22). Thus, a relevant breakthrough of this work is the observation of opposed effects on cellular activation accounted for T-cells derived exosomes in controls and SLE patients. In physiological conditions exosomes could prevent the activation of myeloid cells, but this effect seems to be lost in SLE, where T-cells-derived exosomes were associated with the activation status of monocytes, neutrophils, and lymphocytes, markedly increased in these patients (2). An efficient uptake of T-cells exosomes by other immune cells has been reported, mainly monocytes, and DCs, that in turn can modify their normal functions (22, 23). Accordingly, circulating exosomes isolated from SLE patients have been described as promoters of an inflammatory response, being able to trigger the production of pathogenic cytokines by healthy mononuclear cells (6). Conversely, circulating exosomes from healthy controls in the present study were negatively associated or not correlated to the presence of these cellular subsets, supporting their anti-inflammatory or homeostatic profile in normal conditions (20, 21).

Despite of the T-cell activation in SLE, the counts of CD3^+^-Ex were similar in patients and controls, in contrast to the increased amounts of total exosomes detected in SLE. Nevertheless, this may be due, at least in part, to the lymphopenia present in SLE, since the amount of CD3^+^-Ex for each individual T-cell was significantly increased in patients. Anyway, given that circulating amounts of CD3^+^-Ex were similar in patients and controls, we can hypothesize that qualitative differences could be responsible of these opposite associations. As exosome content depends on the status of parental cells (e.g., activation, differentiation, or senescence), these results may be explained by the altered T-cell subsets usually present in lupus. Thus, the striking reduction of resting T-cells displayed by our SLE cohort, especially in lymphopenic conditions, could be responsible of a limited production of homeostatic T-cell derived exosomes, that contrast with the higher amounts of exosomes derived from senescent and activated cells. Unexpectedly, the frequency of circulating CD3^+^-exosomes in both control and patient groups were associated with the amount of CD4^+^CD28^null^ cells, a cellular subset associated with immunosencescence. These cells were increased and exert a pathogenic role in SLE and other systemic inflammatory conditions (3, 24). The loss of CD28 expression in CD4^+^ T cells is a phenotypic change associated to repeated antigenic stimulation that results in drastic alterations in cell activation, proliferation, and survival (25). Therefore, CD4^+^CD28^null^ cells display potent effector functions such as secretion of inflammatory cytokines, expression of cytotoxic molecules, and resistance to apoptosis and Treg suppression (26–28). But a more surprising result was that CD4^+^CD28^null^ cells displayed similar opposite associations than CD3-exosomes with the leukocyte activated subsets, i.e., positive in patients and negative in controls. Therefore, we can speculate that exosomes derived from senescent T-cells could amplify their pathogenic role in SLE. These results shed light on the regulatory effects of exosomes in pathogenic conditions and suggest the projection of future *in vitro* studies oriented to the identification of molecular mediators revealing the presence of altered composition in SLE exosomes and to evaluate the mechanisms underlying their effects in different cellular subsets.

A remarkable finding from our study was the association between CD3-exosomes and the expression of CD25 in CD4^+^CD28^null^ cells from patients. In physiological conditions, CD4^+^CD28^null^ cells displayed low or no expression of CD25. It has been proposed that the lack of CD25 expression in CD4^+^CD28^null^ T-cells contributes to their reduced ability to proliferate and to the prolonged survival and accumulation of CD28^null^ T-cells in the aging immune system (29, 30). However, we not only observed a significant increase of CD28^null^ T-cells in SLE patients, but also a higher CD25 expression in these cells, supporting that senescent lymphocytes could be activated in pathogenic situations. In fact, these cells can be activated *in vitro* by cytokines or through CD3-stimulation in the absence of costimulatory signals, leading to IL-2 secretion, proliferation, and increased CD25 expression (29, 31). Moreover, the upregulation of pro-inflammatory cytokines and chemokines observed in older compared to younger individuals that includes IL-1, IL-6, TNFα, CCL2, and CXCL10, could be related to the NF-κB transcriptional signature of activated senescent T-cells (32). A novel insight of this work was that CD4^+^CD28^null^ cells could be also activated *in vivo* by type I IFNs. In the control group, serum levels of IFNα were associated with the expression of CD25 in CD4^+^CD28^null^, whereas lupus patients with high IFN-score (IFN^pos^) exhibit an increased proportion of activated CD25^+^CD28^null^ cells, with no differences in the total CD4^+^CD28^null^ subset compared to the rest of patients. These results are in line with the existing literature, since type I-IFNs are well-known promoters of memory cells and they can also provide survival signals for T cells independently of IL-2 or CD28 engagement (33–35).

All these results allow us to hypothesize that the activated status of senescent T-cells in IFN^pos^ SLE patients may represent a new pathogenic mechanism by which type I IFNs could initiate or perpetuate the inflammatory burden. In this setting, CD3^+^-derived exosomes can act as enhancers of this response, given their association with the activated CD25^+^CD28^null^ subset in these patients. Therefore, the inflammatory and immunosenescent milieu found in lupus, hallmarked by sustained type I IFN signaling, may lead to the generation of exosomes with a specific composition and biologic effects which in turn can amplify the abnormal immune response. In line with this, extracellular vesicles from SLE patients exhibit a higher concentration of immunoglobulins, complement components and nucleic acids (like microRNAs) compared to those from healthy controls (36, 37), and these are able to elicit a TLR-dependent inflammatory response (6, 38). The recognition of SLE-exosome components may activate downstream transcription factors, such as NF-κB and IRF3/7, thereby promoting the induction of type I IFNs and other proinflammatory cytokines in various immune cell types (39). According to this, circulating SLE-exosomes have been reported as *in vitro* inductors of IFNα secretion by pDCs activated through endosomal TLR7/9 (38). Then, the IFN signaling can promote the autocrine activation of DCs and their capacity to stimulate T-cells (40), allowing the subsequent release of (pathogenic) exosomes that could reach and activate immune cells at remote sites via blood circulation, thus boosting a vicious circle of immune stimulation in SLE. Taken together, our findings align with the ability of extracellular vesicles secreted by overstimulated T-cells to activate monocytes and induce inflammatory cytokines (41).

In our SLE cohort, most patients (72.6%) exhibit high IFN-score and were characterized by a poor prognostic, thus confirming previous results in systemic inflammatory conditions displaying elevated type I IFN signature (42). Besides the direct protective effect of type I IFNs against viruses, increased IFN-I signaling for a prolonged period may promote aberrant inflammatory responses during bacterial or viral infections, which can be complicated with (hyper)-cytokinemia or even a cytokine storm in severe forms (43). In the same way, in SLE and other chronic inflammatory diseases, sustained IFN-I signaling can promote aberrant inflammatory responses (42). Accordingly, IFN^pos^ SLE patients, in addition to the activation of senescent T-cells, presented high serum levels of several inflammatory cytokines and chemokines, including IL-6, IFNγ, ICAM-1, CXCL10, CCL2, and CCL3, than their IFN^neg^ counterparts. Moreover, in this patient group, CD25^+^CD28^null^ cells were correlated with the activated state of myeloid cells and lymphocytes and with serum levels of CCL3 and IL-17.

Therefore, we can speculate that individuals with elevated CD4^+^CD28^null^ cells and sustained IFN-I signaling may undergo activation of this senescent T-cell subset and secretion of inflammatory mediators, which will be a risk factor for the development of (hyper)-cytokinemia in those with genetics or environmental triggers.

In conclusion, our results draw a picture where CD3^+^-exosomes and activated CD4^+^CD28^null^ lymphocytes could mediate a novel pathogenic mechanism by which type I IFN signaling may promote leukocyte over-activation and secretion of inflammatory mediators in SLE. Hence, interventional studies targeting the type-I IFN pathway have shown clinical benefits in immunopathologies associated to IFN-signatures (44). Therefore, we can hypothesize that the specific content of lupus T-cell derived exosomes could be partially responsible of the immunostimulatory effect underlying the leukocyte activation usually present in these patients, thus making them a promising therapeutic tool in SLE.

## Data Availability Statement

The raw data supporting the conclusions of this article will be made available by the authors, without undue reservation.

## Ethics Statement

The studies involving human participants were reviewed and approved by Regional Ethics Committee for Clinical Research (Servicio de Salud del Principado de Asturias). The patients/participants provided their written informed consent to participate in this study.

## Author Contributions

PL participated in the study conduct, cellular and cytokine quantification, data analysis/interpretation, and manuscript preparation. JR-C contributed to experimental procedures. LC-M contributed to sample collection as well as review of demographic, clinical manifestations, disease activity, and therapy of patients. AS contributed to study design/conduct, data interpretation, and manuscript preparation. All authors contributed to the article and approved the submitted version.

## Conflict of Interest

The authors declare that the research was conducted in the absence of any commercial or financial relationships that could be construed as a potential conflict of interest.
